# Supplementary figures and images for: A viral guide RNA delivery system for CRISPR-based transcriptional activation and heritable targeted DNA demethylation in Arabidopsis thaliana
Source: PLoS Genet. 2020 Dec 14;16(12):e1008983. doi: 10.1371/journal.pgen.1008983 (PMC7769603; doi:10.1371/journal.pgen.1008983)

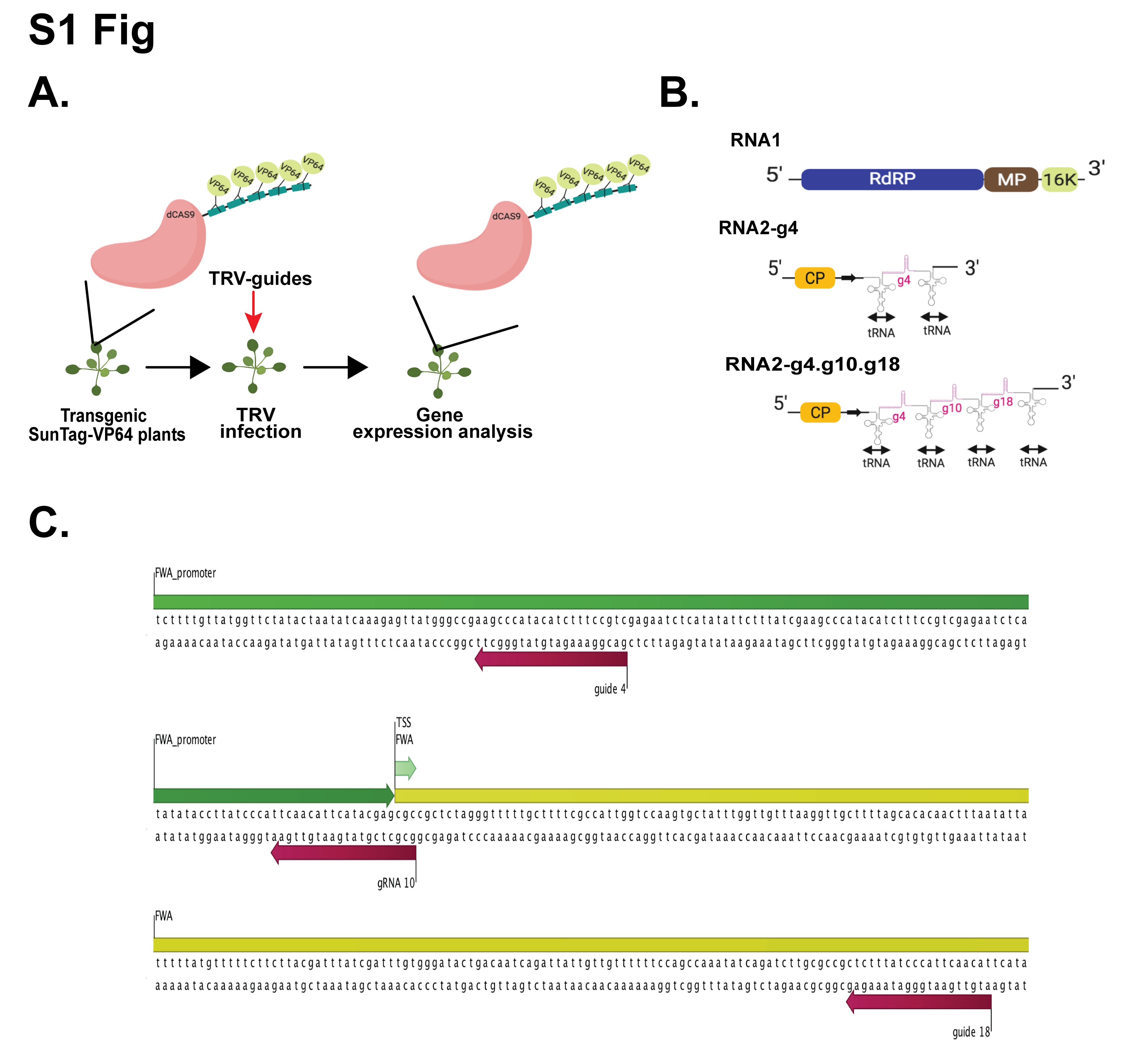

Supplement: S1 Fig — The FWA gene was targeted using guide RNAs delivered to a SunTag-VP64 overexpressing Arabidopsis thaliana line. (A) Schematic representation of the guide RNA delivery process by TRV to Arabidopsis thaliana plants. In the diagram, VP64 is shown as the effector, which is bound to the epitope tail. For targeted DNA demethylation experiments, plants with the SunTag system but with TET1 as the effector was used. (B) Sketch of TRV RNA1 and modified TRV RNA2 with a single guide RNA (guide 4 (g4)) and three guide RNAs (guide 4, guide 10 and guide 18 (g4.g10.g18)) engineered into the genome. TRV RNA1 encodes viral RNA dependent RNA polymerase (RdRP), movement protein (MP), and suppressor of RNA silencing (16K). Guide RNAs were flanked by tRNA sequences to properly process the guide RNAs from the TRV RNA2 genome. Black arrow next to Coat protein (CP) denotes the sgPeBV promoter. (C) Location of guide RNA 4, guide RNA 10, and guide RNA 18 binding sites at FWA relative to transcription start site (TSS). (S1A and S1B Fig were created with BioRender.com.) (TIF) [file pgen.1008983.s001.tif]

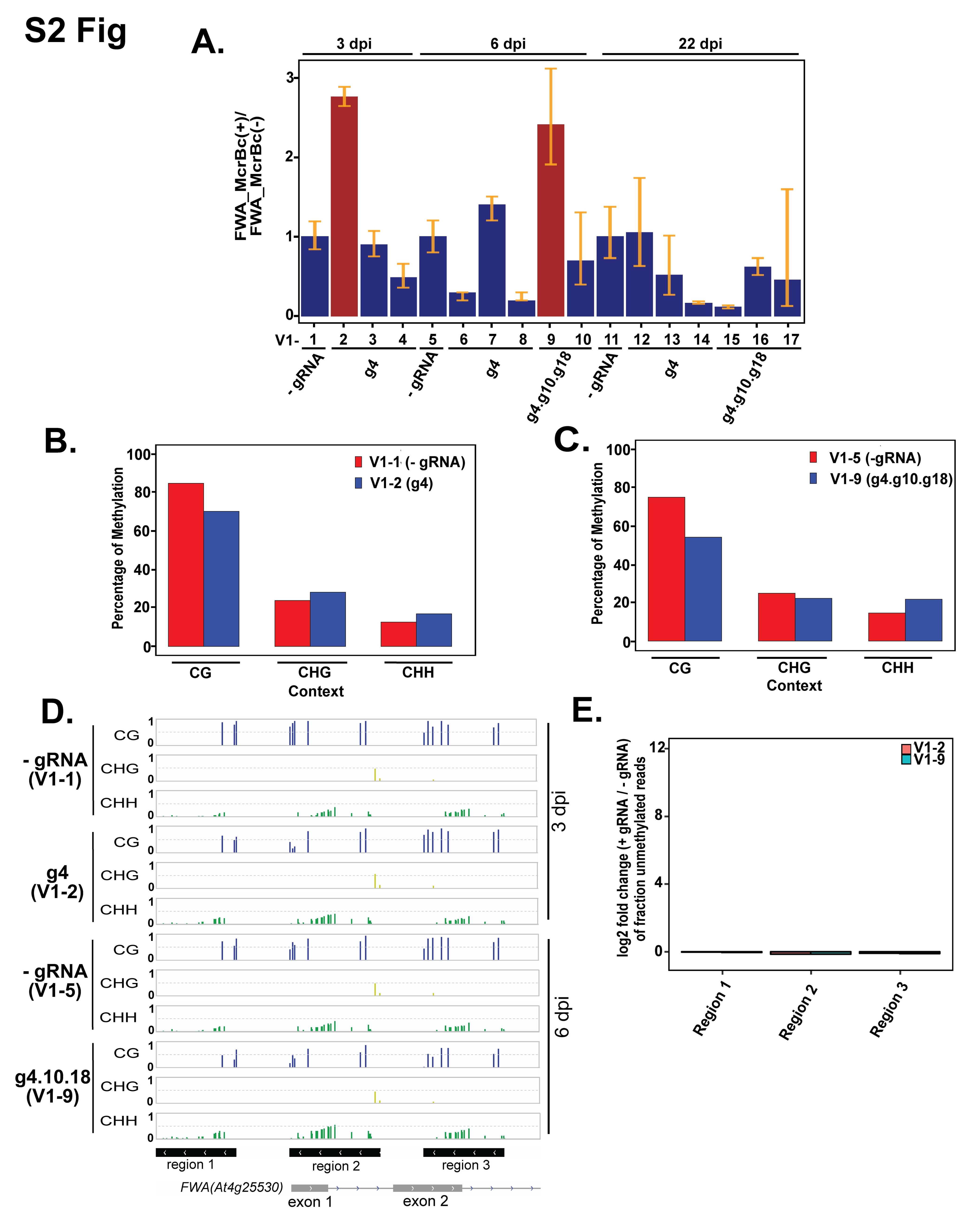

Supplement: S2 Fig — (A) DNA methylation at FWA was analyzed by an McrBC-qPCR based assay for all plants in the V1 generation. Error bars indicate standard deviations (n = 3 technical replicates). (B-C) Bar plots showing average methylation levels in different cytosine contexts at FWA promoter in region 1 (Chr4:13038143 to 13038272), region 2 (Chr413038356 to 13038499) and region 3 (Chr4:13038568 to 13038695) in single guide RNA (V1-2) and multiple guide RNA (V1-9) inoculated plants and their respective controls (V1-1 and V1-5). Only cytosines with at least 5 overlapping reads were used for this analysis. (D) DNA methylation data from bisulfite PCR sequencing, for cytosines in CG, CHG, and CHH contexts, at region 1, region 2, and region 3 of the FWA promoter. An average of 30,000 to 50,000 reads were obtained for each of the three regions. Colors indicate methylation context (CG = Blue, CHG = Yellow, CHH = Green). (E) Data in (D) is displayed as a bar graph of the log2 ratio of the fraction of fully CHH-demethylated reads in +gRNA plants relative to their no guide RNA controls. (TIF) [file pgen.1008983.s002.tif]

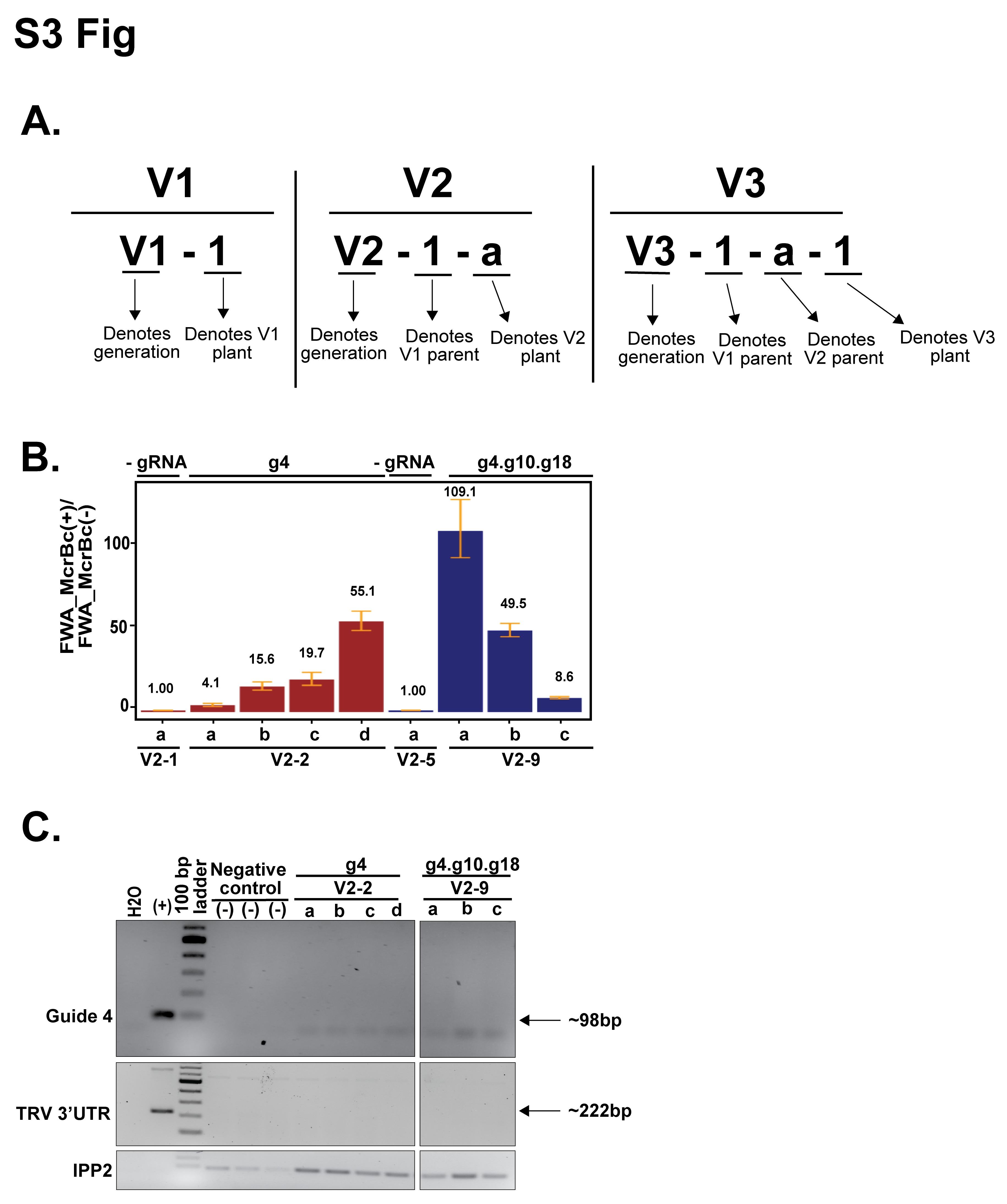

Supplement: S3 Fig — (A) Pattern of naming plants in V1, V2, and V3 generations. (B) DNA methylation at FWA by McrBC-qPCR based assay of plants exhibiting late flowering phenotypes. Error bars indicate standard deviations (n = 3 technical replicates). (C) Agarose gel electrophoresis of reverse transcriptase PCR products amplified to detect guide RNA 4 (98 bp band) and TRV 3’UTR (222 bp band) specific sequences in leaf samples of V2 plants exhibiting late flowering phenotype and their respective controls. H20 = no-DNA negative control, (+) denotes positive control (plasmid containing guide 4 or 3’UTR). Negative controls are RNA from plants that were not inoculated with viruses but were grown side by side at the same time. IPP2 is used as quality control for the DNA sample. (TIF) [file pgen.1008983.s003.tif]

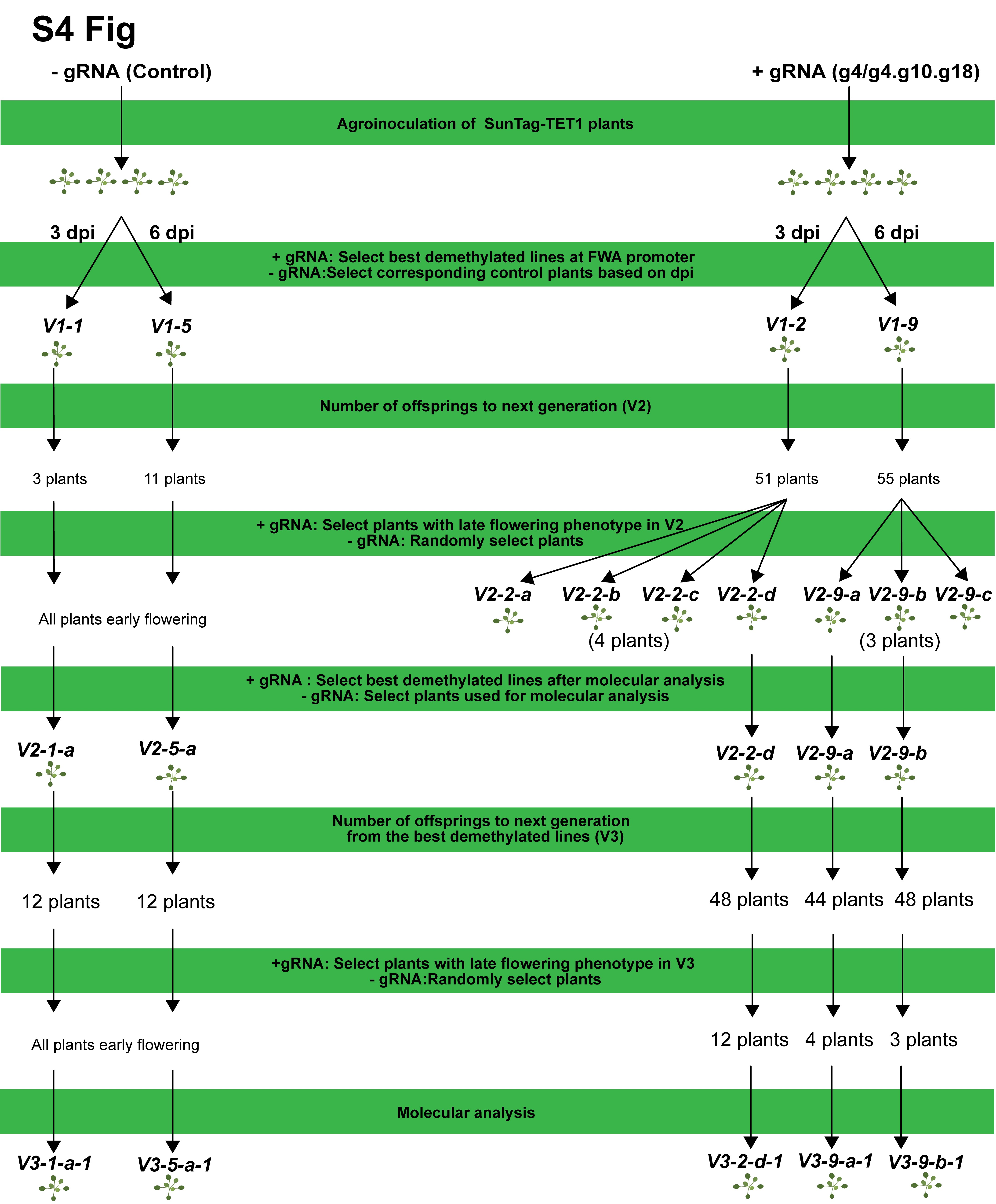

Supplement: S4 Fig — (S4 Fig was created with BioRender.com.) (TIF) [file pgen.1008983.s004.tif]

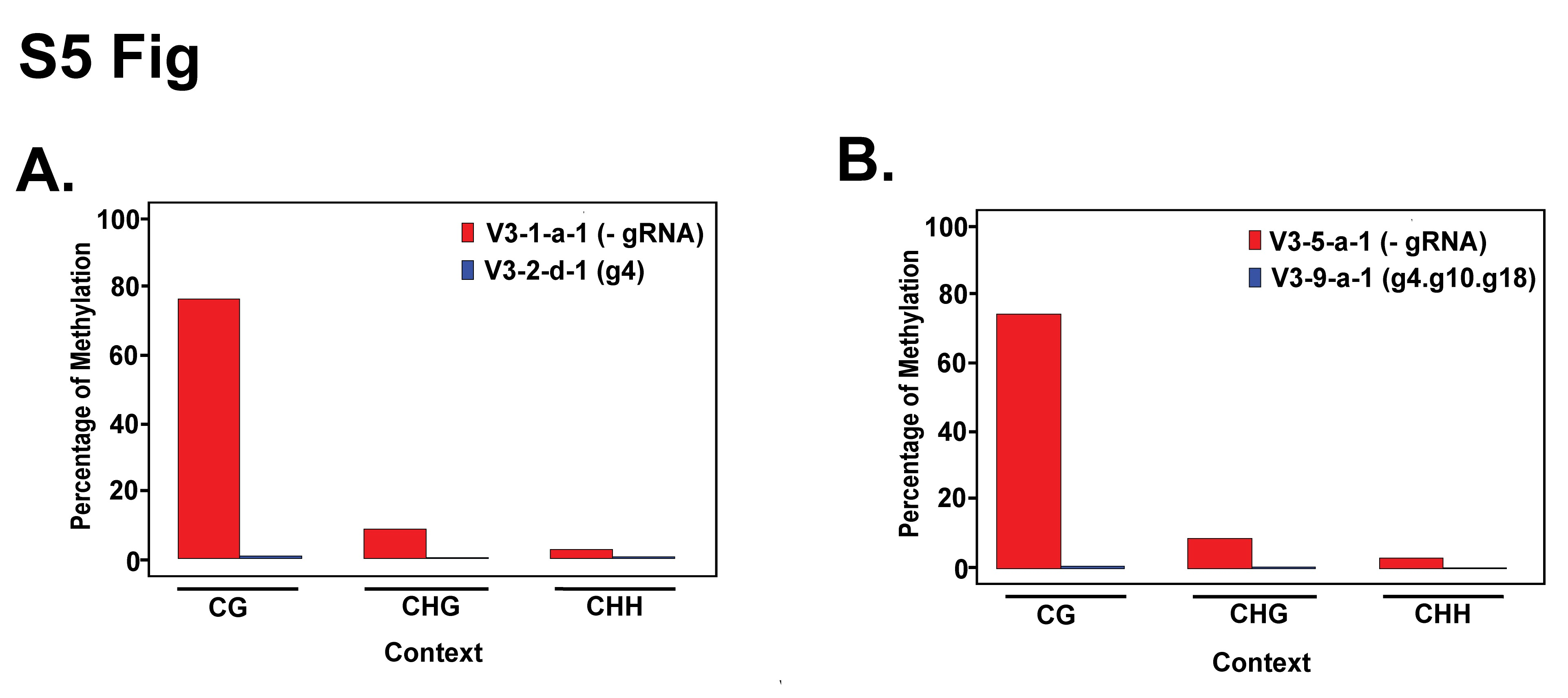

Supplement: S5 Fig — (A-B) Bar plots showing average methylation levels at different cytosine contexts at FWA promoter spanning over 800 bp region (Chr4:13038100 to 13038900) in progeny of single guide RNA (V3-2-d-1) and multiple guide RNA (V3-9-a-1) inoculated plants and their respective controls (V3-1-a-1 and V3-5-a-1). Only cytosines with at least 5 overlapping reads were used for this analysis. (TIF) [file pgen.1008983.s005.tif]

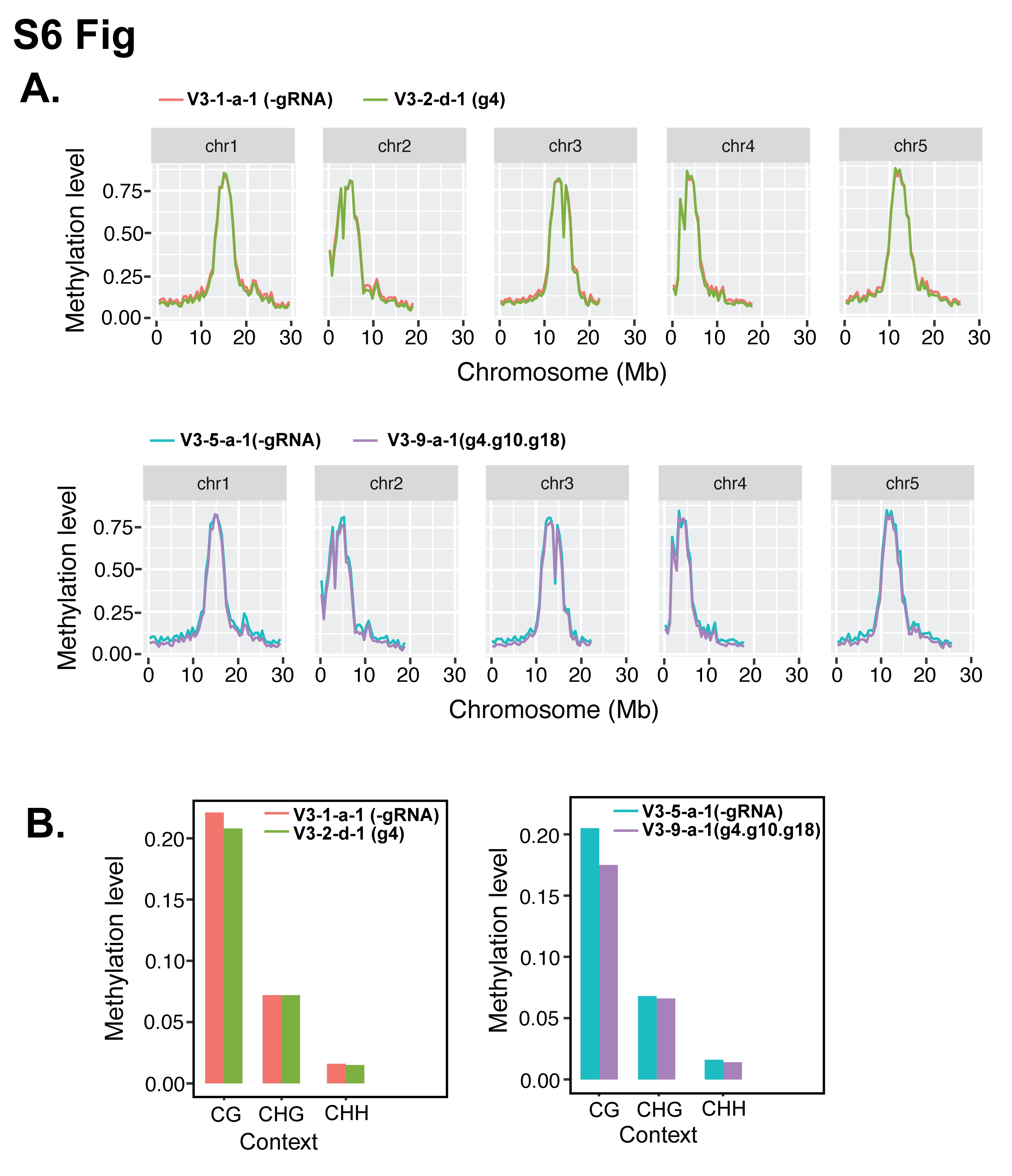

Supplement: S6 Fig — (A) Whole genome CG methylation profile at the chromosome level in V3 plants exhibiting late flowering phenotype compared to their control plants. Metaplots over chromosomes were generated by ViewBS using default parameters. The lines selected for the analysis are shown at the top of the respective plots. (B) Bar graphs showing whole genome methylation percentage in two late flowering plants (V3-2-d-1, V3-9-a-1) compared to their matched controls (V3-1-a-1, V3-5-a-1). (TIF) [file pgen.1008983.s006.tif]

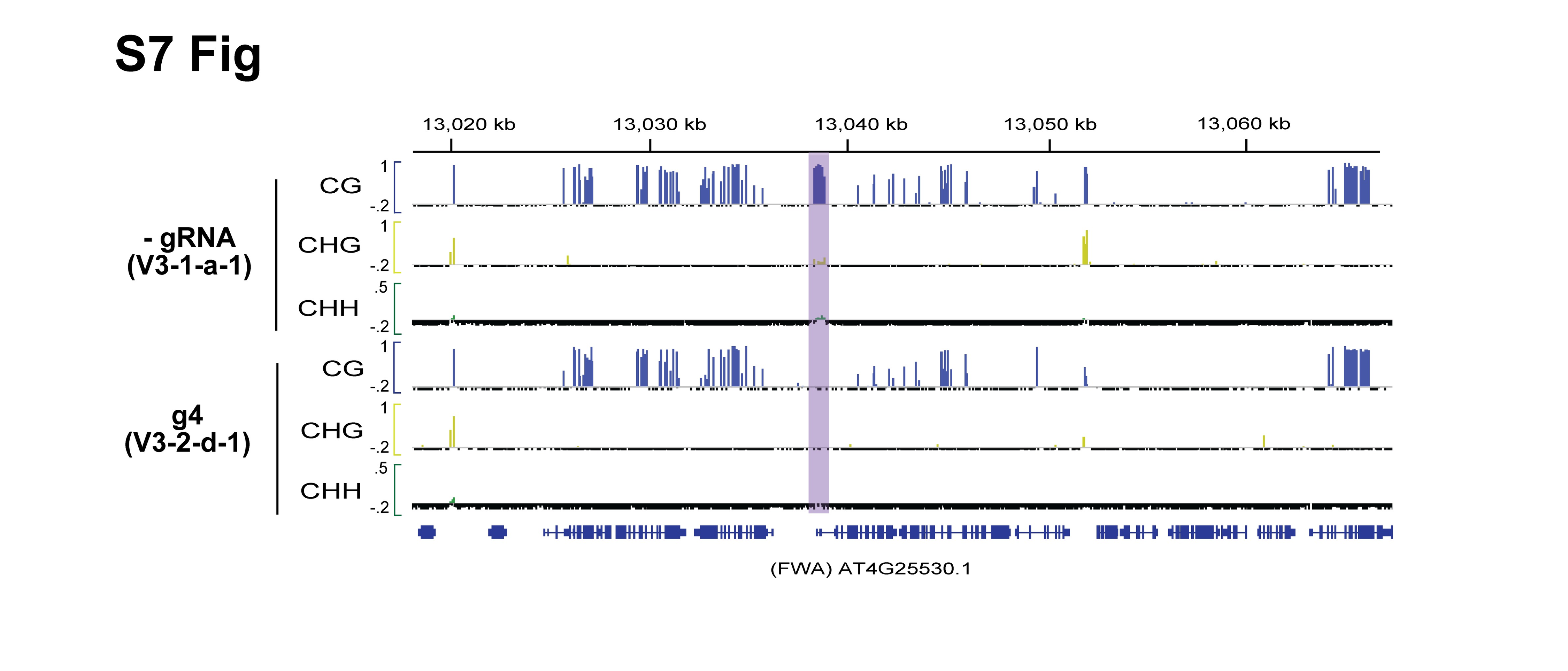

Supplement: S7 Fig — Zoomed out view of CG, CHG and CHH methylation at the FWA promoter and its neighboring genes. (CG = Blue, CHG = Yellow, CHH = Green). Only cytosines with at least 5 overlapping reads are shown. Small, negative values (black) indicate cytosines with 5 or more overlapping reads but no DNA methylation. (TIF) [file pgen.1008983.s007.tif]

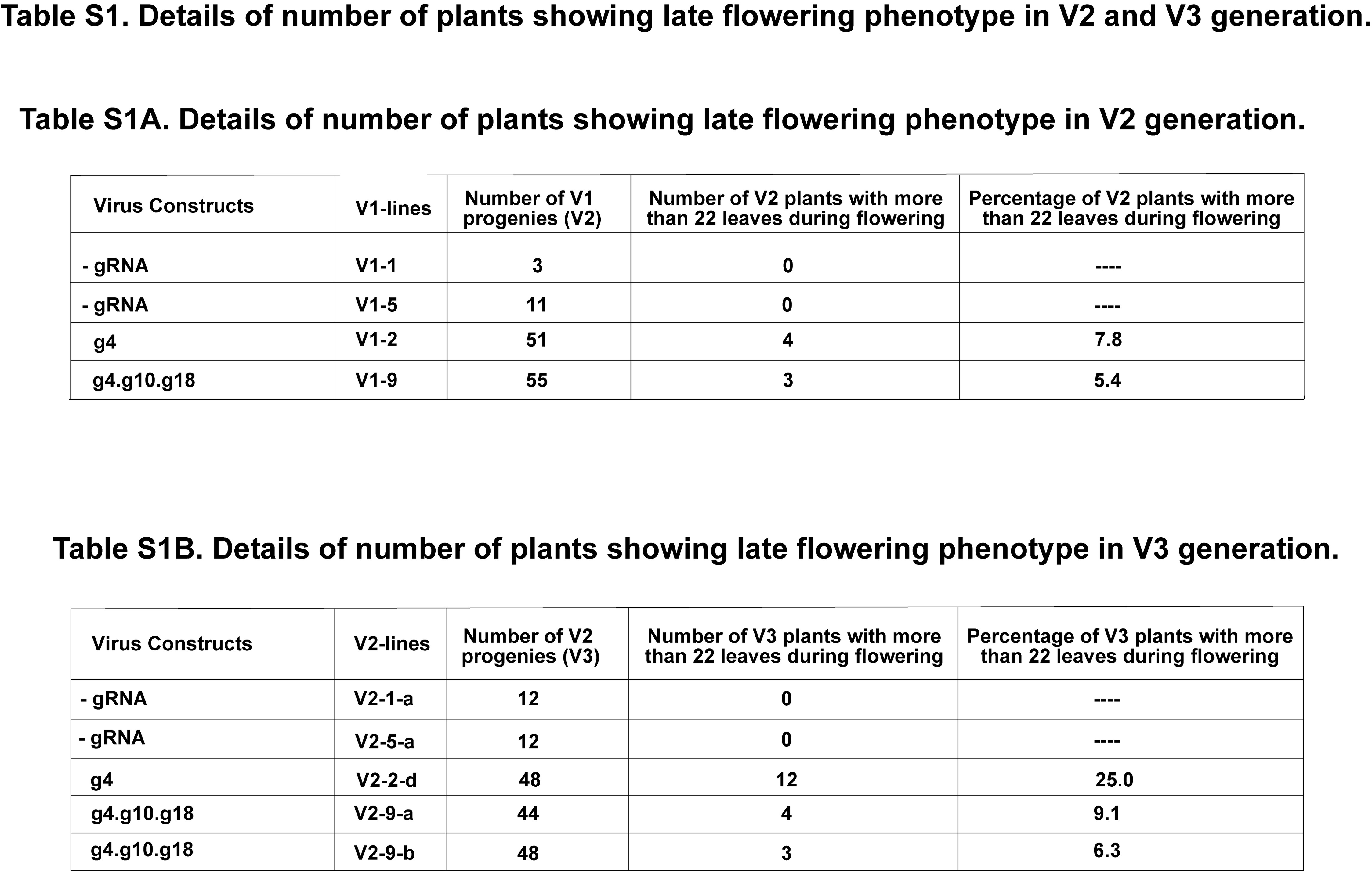

Supplement: S1 Table — (A) Number of plants showing a late flowering phenotype in V2 generation. A late flowering phenotype was determined by counting rosette and cauline leaves at the time of bolting. Plants with more than 22 leaves at bolting were considered late flowering. (B) Number of plants showing a late flowering phenotype in V3 generation. In a previous study, Gallego-Bartolome et al. examined a large number of SunTag-TET1 no guide RNA plants and monitored it for late flowering phenotype. All the SunTag-no guide RNA plants flowered at the same time as Col plants. So, we did not use equal number of SunTag-TET1 no guide RNA plants. (TIF) [file pgen.1008983.s008.tif]

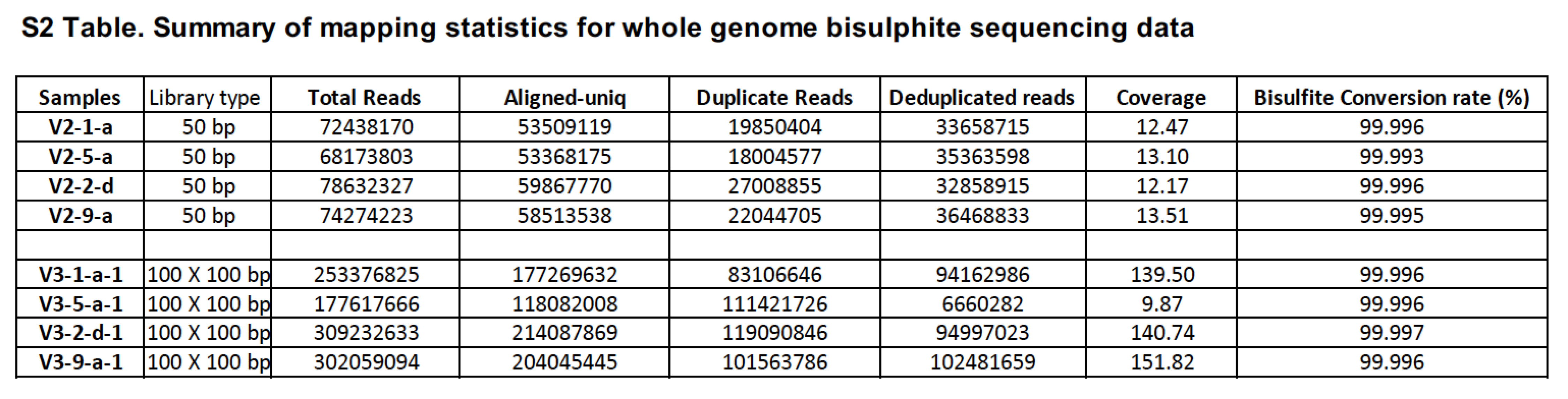

Supplement: S2 Table — The mapping statistics include information about name of the sample (Sample), type of library -paired-end or single-end (Library type), total reads or read pairs (if-paired-end) obtained from sequencing (Total reads), total reads or read pairs that aligned uniquely to the genome (Aligned-uniq), reads removed that aligned to the same position in the genome which can arise due to PCR amplification (Duplicate reads), total reads or read pairs that aligned uniquely and remained after PCR duplicates were removed (Deduplicated reads), coverage and the bisulfite conversion rate. (TIF) [file pgen.1008983.s009.tif]

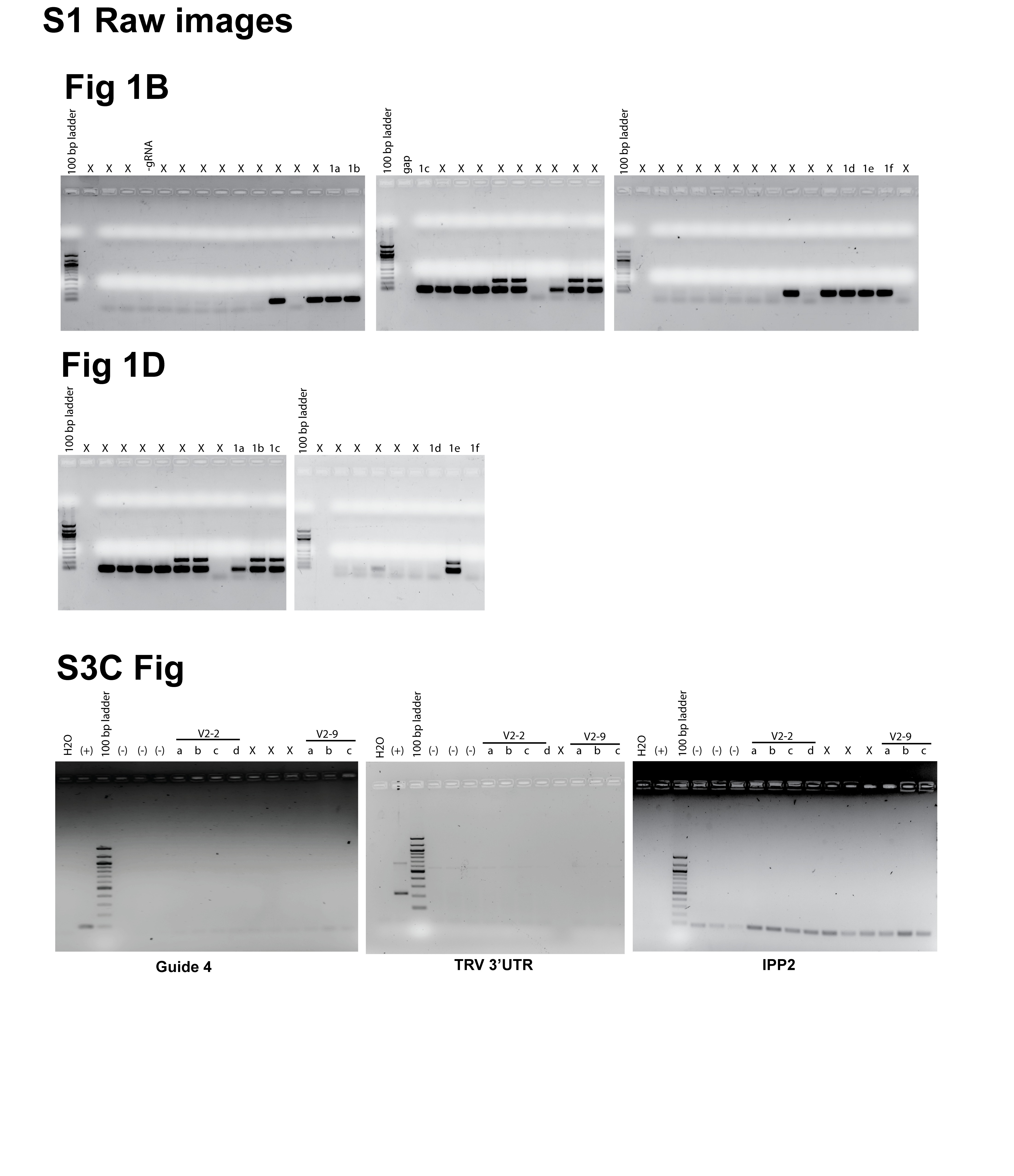

Supplement: S1 Raw Images — (TIF) [file pgen.1008983.s011.tif]
